# Quantifying Fundamental Vegetation Traits over Europe Using the Sentinel-3 OLCI Catalogue in Google Earth Engine

**DOI:** 10.3390/rs14061347

**Published:** 2022-03-10

**Authors:** Pablo Reyes-Muñoz, Luca Pipia, Matías Salinero-Delgado, Santiago Belda, Katja Berger, José Estévez, Miguel Morata, Juan Pablo Rivera-Caicedo, Jochem Verrelst

**Affiliations:** 1 Image Processing Laboratory (IPL), University of Valencia, 46980 Paterna, Spain; 2 Institut Cartografic i Geologic de Catalunya (ICGC), Parc de Montjüic, 08038 Barcelona, Spain; 3 Department of Applied Mathematics, University of Alicante, 03690 Alicante, Spain; 4 Department of Geography, Ludwig-Maximilians-Universität München (LMU), Luisenstr. 37, 80333 Munich, Germany; 5 Secretary of Research and Graduate Studies, CONACYT-UAN, Tepic 63155, Mexico

**Keywords:** vegetation traits, Sentinel-3, TOA radiance, OLCI, Gaussian process regression, machine learning, hybrid method, time series, Google Earth Engine

## Abstract

Thanks to the emergence of cloud-computing platforms and the ability of machine learning methods to solve prediction problems efficiently, this work presents a workflow to automate spatiotemporal mapping of essential vegetation traits from Sentinel-3 (S3) imagery. The traits included leaf chlorophyll content (LCC), leaf area index (LAI), fraction of absorbed photosynthetically active radiation (FAPAR), and fractional vegetation cover (FVC), being fundamental for assessing photosynthetic activity on Earth. The workflow involved Gaussian process regression (GPR) algorithms trained on top-of-atmosphere (TOA) radiance simulations generated by the coupled canopy radiative transfer model (RTM) SCOPE and the atmospheric RTM 6SV. The retrieval models, named to S3-TOA-GPR-1.0, were directly implemented in Google Earth Engine (GEE) to enable the quantification of the traits from TOA data as acquired from the S3 Ocean and Land Colour Instrument (OLCI) sensor.Following good to high theoretical validation results with normalized root mean square error (NRMSE) ranging from 5% (FAPAR) to 19% (LAI), a three fold evaluation approach over diverse sites and land cover types was pursued: (1) temporal comparison against LAI and FAPAR products obtained from Moderate Resolution Imaging Spectroradiometer (MODIS) for the time window 2016-2020, (2) spatial difference mapping with Copernicus Global Land Service (CGLS) estimates, and (3) direct validation using interpolated in situ data from the VALERI network. For all three approaches, promising results were achieved. Selected sites demonstrated coherent seasonal patterns compared to LAI and FAPAR MODIS products, with differences between spatially averaged temporal patterns of only 6.59%. In respect of the spatial mapping comparison, estimates provided by the S3-TOA-GPR-1.0 models indicated highest consistency with FVC and FAPAR CGLS products. Moreover, the direct validation of our S3-TOA-GPR-1.0 models against VALERI estimates indicated with regard to jurisdictional claims in good retrieval performance for LAI, FAPAR and FVC. We conclude that our retrieval workflow of spatiotemporal S3 TOA data processing into GEE opens the path towards global monitoring of fundamental vegetation traits, accessible to the whole research community.

## 1 Introduction

Accurate monitoring of terrestrial photosynthetic capacity is crucial for understanding ecological processes and modelling the responses of vegetated ecosystems to diverse environmental changes [1,2]. Numerous optical sensors are currently available on spaceborne platforms to collect data of the global land surface. In this respect, the European Space Agency (ESA) launched the Copernicus program including the Ocean and Land Colour Instrument (OLCI) aboard Sentinel-3 (S3), which followed the Medium Resolution Imaging Spectrometer on the ESA's ENVISAT platform, for measuring land (and ocean) radiances at high accuracy for diverse environmental monitoring applications [2,3]. Among multiple instruments foreseen to collect data over global terrestrial landscapes in the near future, the ESA “FLuorescence EXplorer" (FLEX) mission is planned to be launched in 2024. FLEX will specifically dedicate to vegetation fluorescence measurements and will partner with the operational Sentinel-3C in a tandem mission [4]. ESA already initiated preparatory studies to develop the ground processing prototype, which eventually will process both incoming FLEX and S3 data streams into a suite of level-1 and level-2 (L2) products accessible for the community [5].

As part of the S3-FLEX L2 processing chain, essential biochemical and biophysical variables will be derived from either FLEX or S3 data streams, or from a combination of both, such as: (1) leaf chlorophyll content (LCC), (2) leaf area index (LAI), (3) fraction of absorbed photosynthetically active radiation (FAPAR), and (4) fractional vegetation cover (FVC) [6,7]. These four vegetation traits not only serve to interpret the recorded fluorescence signal towards estimates of photosynthetic activity, but are also fundamental for mapping and monitoring vegetation dynamics over large areas in space and time [7]. The spectra covered by the OLCI (and FLEX) sensors along the visible and near infrared regions is sensitive to vegetation structure and biochemistry, and therefore for modelling of the four traits [8]. Since LCC is directly involved in the synthesis of biochemical energy, it serves as potential indicator of photosynthetic capacity [9]. LAI corresponds to half the total intercepting area of leaves per unit ground surface area [10]. This variable is strongly related to canopy photosynthesis and evapotranspiration [11] having a key role in the exchange of energy and water between biosphere and atmosphere. Different definitions of LAI are presented in the literature, ranging from effective plant area index [12], which includes the area from all plant organs and assumes random distributions of leaves, to green LAI (GAI, [13]). GAI is probably the most relevant variable to describe the radiation transfer in vegetated canopies and is also frequently used in remote sensing vegetation studies [14]. Though referring mainly to the green plant elements, for sake of simplicity, we use the term LAI throughout this study. Green FAPAR refers to the amount of incoming solar radiation absorbed by live vegetation in the spectral range from 400-700 nm, divided by the total amount of radiation absorbed at the surface [15,16]. One should differentiate between instantaneous values measured during the satellite transit time, and daily averaged values [17]. FAPAR is an important variable commonly used in the modelling of primary productivity [11] and provides an observational constraint on the terrestrial biosphere for simulating vegetation-atmosphere carbon fluxes [16,18]. Moreover, it has proven to be a reliable tool for regional crop yield forecasting when analysing FAPAR time series [19]. FVC corresponds to the fraction of green vegetation as seen from nadir, and is a key biophysical variable reflecting the spatial extent of the photosynthetic leaf area [20,21]. The variable is useful for various agricultural disciplines, ranging from irrigation (e.g., [22]) to yield estimations (e.g., [23]).

Regarding the retrieval of these variables from satellite data streams, most traditional methods relied on parametric regression approaches. These methods assume an explicit relationship between spectral observations (or vegetation indices) and in-field measured variables [24–26]. Parametric regression approaches require only low computational resources. However, they may underexploit available spectral information, such as from S3, as often only two or three bands are implemented. In addition, these few bands can be prone to noise which further limits the genericity and transferability of the established models [27,28]. To overcome drawbacks of empirical approaches, physically based methods have been developed, denominating the implementation of radiative transfer models (RTMs) and their radiometric inversion using cost functions [29–31]. However, these methods are computationally expensive when applied on a per-pixel basis, limiting their applicability for processing large scenes at the continental scale. Data-driven nonparametric nonlinear approaches, such as machine learning regression algorithms (MLRAs), can be a fast and efficient alternative, as shown for S3 data by Verrelst et al. [32]. MLRAs provide adaptive and robust relationships between the variables of interest and spectral signals without knowledge of the underlying data distribution [28]. When it comes to efficient processing of both hyperspectral FLEX and superspectral S3 data into vegetation properties, the so-called hybrid models were evaluated as most promising to achieve fast and consistent retrievals [6]. Hybrid models blend the physical foundation from RTMs with the flexibility of a MLRA [33]. A discussion on its strengths and weaknesses as opposed to other retrieval strategies in operational settings is provided by Verrelst et al. [28,34]. The pursued strategy of hybrid models, for instance applied to processing of FLEX and S3 data streams, is based upon the generation of a training data set using the leaf-canopy RTM SCOPE (Soil Canopy Observation, Photochemistry and Energy fluxes) [35]. In this way, a diversity of vegetation states can be simulated, reflecting actual conditions of the specific location and beyond. The simulated data sets are then used to train Gaussian process regression (GPR) [36] algorithms to build efficient variable retrieval models. While the RTM explores the underlying physical causalities, the MLRA with its universal prediction capability identifies the most relevant (spectral) inputs for inference of a specific output. Hence, hybrid approaches ideally combine the two different paradigms of physical and empirical assumptions, enabling a symbiotic relationship between them [37]. Being a probabilistic MLRA, GPR should be preferred as key retrieval algorithm over others, such as artificial neural networks (ANN), due to its ability to provide associated uncertainty estimates along with the predictions [38].

Presently, the entire S3 OLCI radiance data (L1B) and reflectance data (L2A) stream became readily available to the community, e.g., through the Copernicus Hub [39]. Along with it, operationally derived vegetation products are offered by the Copernicus Global Land Service (CGLS), such as the OLCI terrestrial chlorophyll index (OTCI, [24]), indicating canopy chlorophyll content, and the OLCI global vegetation index (OGVI [40]), currently renamed to GI-FAPAR. The S3 OLCI green instantaneous FAPAR product was recently evaluated by Gobron et al. [41]. The algorithm uses a physically based approach simulating reflectance of different land covers, which allowed to compute green FAPAR from top-of-atmosphere (TOA). However, most of these vegetation products have been developed independently of each other and may therefore lack physical consistency. In addition, although quality flags are provided, the products miss associated uncertainty estimates.

With the ambition to provide key vegetation products in a physically consistent and efficient way, De Grave et al. [6] developed hybrid models for LCC, LAI, FAPAR and FVC exploring OLCI surface reflectance and FLEX sensor data synergies. A prototype version of the hybrid retrieval models was recently validated and applied to single S3-OLCI tiles [42]. Hybrid models process single images relatively quickly. However, when it comes to processing at a continental scale, computationally efficient solutions have to be sought. Moreover, essential preprocessing steps, such as selecting and preparing S3 tiles from the Copernicus data hub, add up to computation time. In addition, imposed restriction to the data hub can be a substantial bottleneck even for fully automated processes. Consequently, to achieve dynamic processing of a vast amount of S3 data, there is the need to migrate towards cloud-computing platforms. Specifically, the Google Earth Engine (GEE) emerged as an appealing high-level processing platform allowing cloud-based computations at planetary scale for satellite data [43]. Multiple studies have been conducted using GEE for diverse applications (e.g., [44–46]), but only few investigated the potential of GPR models into the GEE environment [47].

Recently, a general adaptation of GPR formulation to a parallel processing framework and its integration in GEE was proposed. For this purpose, Pipia et al. [47] reviewed the standard GPR regression formulation to achieve a factorization suitable for parallel computing. However, when it comes to processing of S3 scenes, currently only TOA L1B data is provided by the GEE platform. This implies that modeling approaches need to be upscaled from top-of-canopy (TOC) reflectance to TOA radiance data, thereby taking atmospheric effects into account. Nonetheless, this challenge also opens opportunities towards interactive on the fly processing of the four fundamental vegetation traits over wide areas, e.g., at the continental scale. The feasibility of using TOA data from the Sentinel-2 multispectral instrument for mapping of crop traits was recently demonstrated by Estévez et al. [48] and Estévez et al. [49].

Altogether, the main objective of this work was to develop a workflow for the operational retrieval of four essential vegetation traits (LCC, LAI, FAPAR and FVC) from Sentinel-3 top-of-atmosphere data directly in the Google Earth Engine environment. To achieve this, we optimized the training of GPR models to run in GEE while reaching acceptable accuracy. A threefold evaluation strategy was pursued to assess the quality of the obtained vegetation traits: (1) temporally, using time series reference products, (2) spatially, in form of variable reference maps, and (3) directly, using interpolated in situ data for a variety of land cover types.

## 2 Materials and Methods

To enable running GPR models in the GEE platform, the implemented workflow started with training and tuning, while the models were built accounting for a diversity of conditions in space and time. The pursued strategy to obtain a “chain of confidence” for generating series of vegetation traits from OLCI L1B data at 300-m resolution included five main processing steps:

1. upscaling of TOC-RTM simulations to TOA radiance;
2. training retrieval algorithms for establishing trait specific S3-TOA-GPR-1.0 models;
3. running the S3-TOA-GPR-1.0 models in GEE at European continental scale;
4. generating of time series profiles;
5. evaluating model estimates and associated uncertainty for different variables and sites.

**Figure 1:**
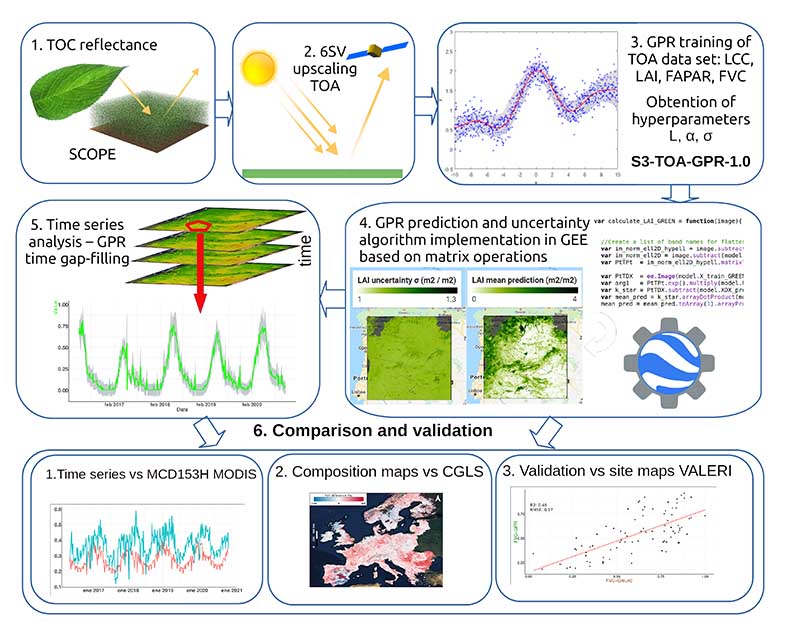
Retrieval workflow for derivation of LCC, LAI, FAPAR and FVC, and S3-TOA-GPR-1.0 model implementation in GEE for mapping, time series generation as well as validation strategies.

Figure 1 summarizes the proposed workflow and the following subsections describe all steps in detail. We start with the used RTM and the training data set generation (Section 2.1). Subsequently, the mathematical background of the GPR models is given (Section 2.2), followed by the vegetation traits mapping and time series analysis (Section 2.3). The specification of the TOA S3-OLCI imagery, provided by the GEE platform and the dedicated regions are detailed in Section 2.4. Finally, Section 2.5 introduces the validation data sets and corresponding processing strategies.

### 2.1 Radiative Transfer Modeling and Training Data Set Generation

For the purpose of our study we employed the RTM SCOPE (v1.7) [35], being a vertical (1D) integrated radiative transfer and energy balance model. To generate TOC reflectance, SCOPE requires, among others, the definition of leaf, canopy, soil and geometry variables and their respective ranges (e.g., LCC, LAI, soil moisture).

FAPAR was calculated as the ratio between the downward direct and diffuse photosynthetically active radiation (PAR, 400-700 nm) and upward fluxes of PAR, as calculated in SCOPE [35]. FVC is obtained empirically from the gap fraction (P) at nadir, by the expression defined in De Grave et al. [6] as follows in Equation (1):

(1) P=exp(−kxLAI)

where *k* is the extinction coefficient. Given this relation, we can obtain FVC in Equation (2) as:

(2) FVC=1−P

The input variable values, ranges and distributions are summarized in Table 1. The Gaussian distribution was used for those variables with expected mean and standard deviations naturally occurring, meanwhile uniform was used for those variables to be predicted. Also, geometrical variables were defined as uniform [50]. In order to provide variability of conditions across latitudes and seasonality, we considered sun zenith angle values between 20° and 40° and observation zenith angles between −10° and 10°. The resulting data set in the size of 1000 samples covered a wide variability of vegetation states, as also applied in previous works [51,52].

Based on the reference TOC OLCI models presented by De Grave et al. [6], we prepared an alternative version specifically adapted to process TOA radiance data. To do so, upscaling with an atmospheric RTM was required. We chose the atmospheric RTM Second Simulation of a Satellite Signal in the Solar Spectrum-vector (6SV) [53] to create a spectral database of atmospheric transfer functions, which were coupled with the established SCOPE training data set. The coupling process was largely automated with the so-called Atmospheric Lookup table Generator (ALG) software [54] and the TOC2TOA toolbox [33] included in the scientific Automated Radiative Transfer Models Operator (ARTMO, Verrelst et al. [55]) software framework, assuming that the land surface is Lambertian, according to the formulation of Guanter et al. [56]. The basic parametrization used for 6SV (v2.1) in this work is detailed in Table 2. The variable ranges of the atmospheric models were chosen according to prior studies [48,49] and should reflect generic and globally valid ranges [54]. The SCOPE simulated training data set was then mixed with additional 1000 samples of spectra extracted from real S3 scenes. The spectra were selected from different points dispersed over Europe and for different seasons, including bare soil and vegetated areas on different proportions as observed from external products, such as from the Moderate Resolution Imaging Spectroradiometer (MODIS)—MCD15A3H [57]. The inclusion of these external spectra was required to provide a realistic training data set, reflecting the actual spectral conditions of the satellite scenes and thus to guarantee high mapping quality for heterogeneous land surfaces. The final training data set was composed of a total number of 2000 samples.

Finally, we trained the GPR algorithms based on a randomly selected data set at the size of 250 different combinations of variables and corresponding TOA radiance, using the ARTMO's MLRA toolbox [58]. Note that the memory restriction in GEE do only allow the implementation of GPR models with a feasible size. Additionally, as a GPR model scales cubically with the size of the training data set [36], it bears the consequence that the sample size has to be sufficiently small. The obtained retrieval models for LCC, LAI, FAPAR and FVC were then subsequently validated against the leftover simulations (N = 1750) using the goodness-of-fit metrics coefficients of determination (R^2^), root mean squared error (RMSE) and the normalized RMSE (NRMSE). The final models, named as "S3-TOA-GPR-1.0", were then imported in GEE as described in the next sections.

### 2.2 Gaussian Process Regression Approach

We followed the regression methodology described in Rasmussen and Williams [36] to build our S3-TOA-GPR-1.0 retrieval models. Henceforth, we provide the mathematical definitions to assess the internal consistency of such models together with the predictions, for the direct implementation into GEE. In short, GPR approximates the relationship between input samples $\text{x}\in {\mathbb{R}}^{D}$ and output observations y∈ℝasy=f(x)+ϵ, being ε an additive Gaussian noise with zero mean and variance $\sigma_{n}^{2}$ and *f* (**x**) a Gaussian distributed random vector with zero-mean and Covariance matrix **K**(**x**, **x**). It is worth recalling that the Covariance matrix accounts for the similarity between pairs of input samples **x**_*i*_ and **x**_*j*_ using a kernel function *k*(**x**_*i*_, **x**_*j*_) for sample distance quantification. Among the multiple kernel functions available, the Asymmetric Square Exponential function has been demonstrated to be efficient for vegetation modelling from Earth observation data [59], being defined as:

(3) $$k\left({\text{x}}_{i},{\text{x}}_{j}\right)=\sigma_{s}^{2}\exp\left(-\frac{1}{2}\overset{D}{\underset{b=1}{\sum }}{\left[\frac{{\text{x}}_{i}(b)-{\text{x}}_{j}(b)}{\sigma_{b}}\right]}^{2}\right),$$

where $\sigma_{s}^{2}>0$ is the output variance while *σ_b_* is related to the relevance of dimension (or band) *b* in the prediction process: the higher *σ_b_*, the lower informative content of *b*. Being $\theta =\left\{\sigma_{s}^{2},\sigma_{1},\ldots ,\sigma_{\text{D}},\sigma_{n}^{2}\right\}$, the probability of the observations given the model’s hyperparameters *p*(***y***|***x***, ***θ***) is given by the marginal likelihood over the function values *f* [36], so that its maximization provides directly the optimum value of ***θ*** to be used for **K**(**x**, **x**) estimation. This procedure is usually referred to as GPR training [36,47]. Afterwards, the prediction of *y* for a new input vector ***x***_∗_ is obtained along with its uncertainty as:

(4) $$\begin{array}{r} f\left(x_{*}\right)=k_{*}^{T}{\left(K+\sigma_{n}^{2}I_{N}\right)}^{-1}y\\ \sigma_{f}^{2}\left(x_{*}\right)=c_{*}-k_{*}^{T}{\left(K+\sigma_{n}^{2}I_{N}\right)}^{-1}k_{*} \end{array}$$

where *N* is the number of training samples, ***k***_∗_ = [*k*(***x***_∗_, ***x***_1_),…, *k*(***x***_∗_, ***x**_N_*)]^*T*^ contains the similarity between ***x***_∗_ and the training input information, ***y*** = [*y*_1_,.., *y_N_*]^*T*^ is the training output, and $c_{*}=k\left(x_{*},x_{*}\right)+\sigma_{n}^{2}.\;$.

An alternative factorization of Equation (4), which has been used for our prediction, is summarized next. First, we train the model for hyperparameter optimization and calculate ***K***.

Then, we obtain the low-triangular matrix ***L*** from the expression $K+\sigma_{n}^{2}{\text{I}}_{\text{N}}$ using the Cholesky factorization and calculate the vector ***α*** as :

(5) $$\alpha =L^{T}\backslash (L\backslash y)$$

where the symbol \ denotes the linear equation system solver operator. The vector ***a*** is formed by a set of weight coefficients assigned to each of the training samples, and depends on both ***L*** and the training observations ***y***. The final estimation of the modeled output is calculated by means of Equation (5) as:

(6) $$f\left(x_{*}\right)=k_{⋆}^{T}\alpha .$$

Finally, defining *v* = *L* \ *k*_⋆_ we calculate the uncertainties of our model as [36]

(7) $$\sigma_{f}^{2}\left(x_{*}\right)=k\left({\text{x}}_{⋆},{\text{x}}_{⋆}\right)-v^{T}v$$

where *k*(***x***_⋆_, ***x***_⋆_) indicates the kernel function calculated at the new input ***x***_⋆_.

In practical terms, to implement these expressions in the GEE environment, the following steps were introduced by Pipia et al. [47]: (1) expanding the formulation of standard GPR, (2) aggregating all terms independent of pixel's spectral information that can be precalculated to avoid repeating cumbersome operations for each pixel, (3) manipulating data using image datatype format before moving to array data type, (4) implementing GPR into a matrix algebra formulation, and (5) converting the results back to image format adding coordinates information, mandatory for mapping purposes. In the present work, we extended the algorithm in order to provide the uncertainties (*σ*) through Equation (7)

To fit our GPR models, we generated a collection of TOA radiance spectra (***x*** vectors) together with the corresponding vegetation properties samples (**y**).

### 2.3 Generation of Vegetation Traits Maps and Time Series

The mathematical algorithm described in Section 2.2 was implemented in GEE by filtering the S3-OLCI scenes intersecting our study areas for selected date ranges. We generated composed monthly averaged maps by applying our method on monthly averaged radiances as described in Equation (8):

(8) $$f(\bar{x})∼GP\left(m(\bar{x}),k\left(\bar{x},x^{\prime }\right)\right)$$

being *x̄* the mean of radiances for our interest interval of time, defined by pixel.

We tested the similarity of these results with respect to those calculated as means over single scenes, see Equation (9). We evaluated over 100 points differences between results calculated from both radiances means vs. single scenes averages and we obtained a deviation mean of 5.68%. The differences on found results can be explained by the nonlinear nature of GPR models. We chose the radiances mean approximation due its advantages on performance, as the algorithm is implemented in only one step against the multiple steps needed in the other case. Furthermore, by this approach, we use directly stable means as inputs, avoiding biases due to anomalous conditions found on specific dates.

(9) $$\bar{f}(x)=\sum^{n}f\left(x_{n}\right)/n$$

Regarding the input data preprocessing, we applied “Bitwise“ operation in order to filter out bright pixels as a preliminary cloud pixel classification, and also mask inland water pixels. We used the provided quality flag band for the OLCI L1B product to accomplish this action.

With the purpose of exploring the temporal behaviour of the vegetation traits, we generated spatially averaged time series for a collection of S3-OLCI scenes (April 2016-November 2020). Then we filled temporal gaps by applying the GPR algorithm to the time dimension in GEE. This approach provides smoothness for the analysis of functions over time and enables comparison among different sources of data by reconstructing unavailable values. To mitigate the most important limitations of GPR time series gap-filling caused by high memory/computation time requirements and repetitive processes, we pursued the approach by Belda et al. [60]. This study demonstrated that reliable gap-filling can be achieved by making use of precalculated hyperparameters (parameter's length scale, variance and noise level) which tremendously accelerates the training stage of the GPR algorithm (90 times faster than the standard GPR estimations).

For all tested vegetation variables (LCC, LAI, FAPAR and FVC), the performance of using these global hyperparameters for time series generation over any crop type was only degraded between 2% and 7% compared to using the conventional GPR per-pixel optimization. On that basis, we assumed the validity of trait dependent global hyperparameters also to study the diverse vegetation types included in our Copernicus land cover (CLC) test areas (see Section 2.4). We applied the same approach to interpolate uncertainties over time, obtaining data series with deviations in respect to the expected prediction boundaries. To avoid memory problems when applying the algorithm to the entire S3-OLCI data collection, we divided the process into multiple steps by iterating over monthly data ranges.

### 2.4 Satellite Data & Demonstration Case Studies

The optical input data used for processing into vegetation traits comes from the L1B Earth Observation Full Resolution (EFR) product, measuring radiances from OLCI onboard Sentinel-3A (S3A) and Sentinel-3B (S3B). S3-OLCI provides measurements over 21 bands ranging from 400 to 1020 nm with band widths between 7.5 and 40 nm [61] and a spatial resolution of 300 m.

The temporal frequency of the images intersecting the study areas varied between two days when only S3A was in orbit to one day for observation after the launch of S3B on 25 April 2018. The collection of images used for this work ranged from 20 April 2016 until 20 November 2020. Since after this date anomalous values for the bands 1 and 10 were encountered, we decided to exclude these dates from our analysis.

**Figure 2:**
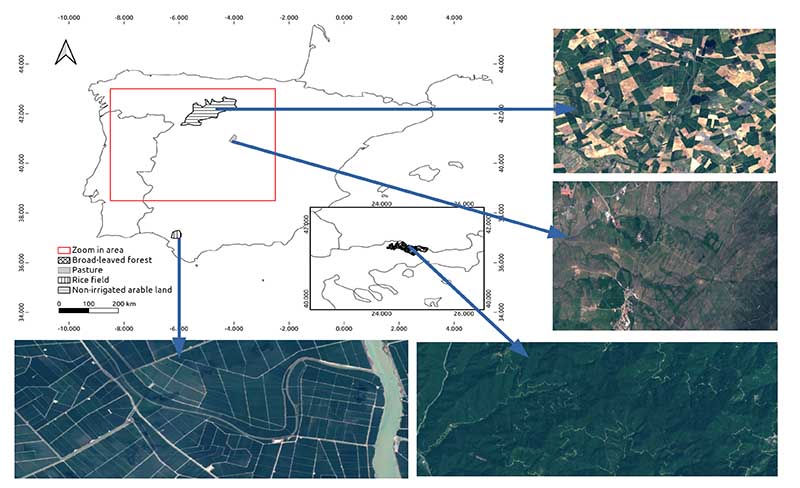
CLC areas used for time series analysis. For more comprehensive insights, RGB captures were taken from Sentinel-2 scenes with GSD of 20 m, at different dates. Counterclockwise: rice field, broad-leaved forest, pasture land and non-irrigated arable land.

To demonstrate the spatiotemporal mapping capability of the S3-TOA-GPR-1.0 retrieval models in GEE, we applied them to the TOA S3-OLCI catalogue over diverse areas in Europe. Monthly averaged maps were generated over all the EU countries and territories of the British Isles. Time series were produced for homogeneous agricultural and forest land covers (Figure 2) according to the CLC classification of the Copernicus program [62], including non-irrigated arable land (NIAL), rice fields (RF), pastures (P) and broad leaf forest (BF1). The extension of the targeted surfaces varies from a minimum of 22,793 ha in the case of pastures to a maximum of 891,098 ha for non-irrigated arable lands. The sites were selected based on the following criteria: (1) extension: to evaluate the capabilities of the models to provide meaningful spatial information at large scale; (2) availability of data: the sites should secure sufficient data availability, hence avoiding locations in northern latitudes with a relatively high percentage of cloudy days; and (3) geographical diversity: the locations have to be geographically dispersed covering different ecosystems across Europe. To identify the locations fulfilling these requirements, we explored the Corine land cover vector database (version 2020).

### 2.5 Validation Data Sets and Strategies

To evaluate retrieval and mapping performance of the S3-TOA-GPR-1.0 models, we compared our estimates over the spatial and temporal domains against analogous products. Additionally, a direct validation with data coming from in situ campaigns was performed. Table 3 provides an overview of the three explored data sets with employed algorithms and validation strategies.

The first data set comes from the NASA Earth Observing System Data and Information System (EOSDIS), the so-called MODIS collection MCD15A3H [57]. The retrieval algorithm is based on the empirical relationship between surface reflectances at 648 nm (red) and 858 nm (infrared) and LAI and FAPAR. The algorithm uses a lookup table (LUT), generated using a three-dimensional (3D) radiative transfer equation [63]. The MODIS MCD15A3H data were directly processed in GEE, as it takes part of the catalog in the cloud. We used the collection of LAI and FAPAR every 4 days, and computed the average of pixels inside the CLCs per date, allowing to generate time series representative of the whole areas.

The second data set was provided by the Copernicus Global Land Service (CGLS) products collection [64]. It includes composition maps gap filled over different time windows (10 days, one month and other periods) of LAI, FAPAR and FVC at a spatial resolution of 300 m. The CGLS data sets were downloaded from the official website: https://land.copernicus.eu/global/themes/vegetation (accessed on 7 June 2021). The estimation of the variables was achieved in a two step process as described by Fuster et al. [64]. In a first step, PROBA-V and OLCI TOA data were converted to TOC reflectances using the Simplified Method for the Atmospheric Correction (SMAC, [65]). Next, daily estimates of LAI, FAPAR and FVC were obtained from these TOC reflectances using pre-calibrated ANN retrieval models. In a second step, smoothing techniques and gap-filling were applied to provide stable maps over different time windows. To perform the comparison, we computed difference maps against the three biophysical variables (LAI, FAPAR and FVC) delivered by CGLS and the corresponding S3-TOA-GPR-1.0 retrieval models. LCC was not included in this analysis as no comparable product was found in the available catalog on the CGLS site, and neither in GEE. The time windows used for the composition maps ranged from 4 March to 19 November 2019. Hence, the comparison was performed over the widest possible time period in this year.

Third, we explored the Validation of Land European Remote Sensing Instruments (VALERI) database (http://w3.avignon.inra.fr/valeri), accessed on 29 September 2021. VALERI is a vast in situ data collection over different campaigns and countries, providing processed maps of interpolated variables for direct validation of grid products [66–68]. The interpolation maps were produced using an empirical transfer function between spectral data of high spatial resolution, such as the SPOT (Satellite Pour l'Observation de la Terre) HRVIR (High-Resolution Visible and Infrared) instrument, and corresponding in situ ground measurements, as detailed by Baret et al. [66]. The VALERI campaigns covered crops, diverse forest types (boreal, deciduous, pine and Mediterranean), grasslands as well as mixed categories. Ideally, S3-OLCI images should be used to validate our products against VALERI in situ data. However, as these campaigns took place before the S3 launch in 2016, we used data from the Landsat ETM (Enhanced Thematic Mapper) mission to process vegetation products for comparison with the VALERI data set. The Landsat data were spectrally resampled to simulate the OLCI spectral bands by using a linear interpolation method. Subsequently, a filter convolution was applied to fit the spectral signal according to the relative spectral response (RSR) of the OLCI bands. Hereby it was only possible to reconstruct 18 out of the 21 bands of OLCI, as the first three bands are beyond the limits of Landsat ETM. These tree bands are located in the blue visible wavelength region and thus highly affected by aerosol effects. Nonetheless, as this spectral region plays a minor role for optical vegetation properties, the exclusion of these bands does not reduce significantly the performance of our models.

Considering that only 18 bands of OLCI could be resampled from Landsat ETM, a substitute S3-TOA-GPR-1.0 model for LAI, FAPAR and FVC based on 18 bands was developed for the comparison. In addition, we only selected those pixels labeled with the highest quality flag. The pixels were then spatially resampled to correspond to the S3-OLCI images scale with ground sampling distance (GSD) of 300 m. A similar validation method is reported by Camacho et al. [69]. Table A1 gives an additional overview of the used VALERI campaigns details.

Note that for all validation cases, FAPAR estimates corresponded to the instantaneous values acquired at the time of the satellite overpass. This value can be considered as reasonable approximation of the daily integrated value [17].

## 3 Results

### 3.1 Theoretical Performances of the S3-TOA-GPR-1.0 Retrieval Models

**Figure 3:**
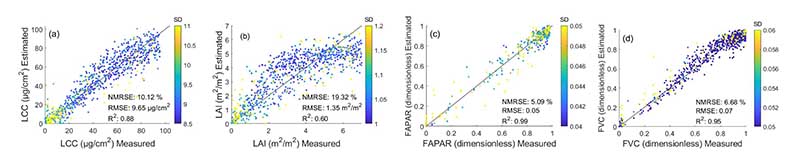
Theoretical validation of trait specific S3-TOA-GPR-1.0 retrieval models: LCC (a), LAI (b), FAPAR (c), and FVC (d) using the simulated SCOPE-6SV database (1750 samples).

The theoretical performances of the S3-TOA-GPR-1.0 retrieval models were evaluated over a subset of the simulated SCOPE-6SV database (75% of full data pool). The results are illustrated in Figure 3, and suggest consistency for all variables with R^2^ ranging from 0.60 (for LAI) to 0.99 (for FAPAR). In addition, relative error measures indicate a very high retrieval accuracy with NRMSE of 19.32% for LAI and NRMSE of 6.68% for FVC. In the case of LAI, the well-known saturation effect occurred [70] for values larger than 4 m^2^/m^2^. The effect was explained with the nonlinear relation between LAI and reflectance for larger LAI values, when the signal becomes less sensitive to canopy structure effects [71]. The other three variables provided higher consistency over their corresponding values ranges, and in case of LCC and FAPAR, we can observe a linear relationship. However, residuals of all four variables are not homoscedastic or normally distributed. In particular for LCC, the larger spread of residuals at higher values suggests heteroscedasticity. This may limit the prediction capacity of the models, depending on the observed variable ranges.

The obtained results provided sufficient confidence for the subsequent implementation of the models in GEE. Hence, the S3-TOA-GPR-1.0 models were applied to the S3-OLCI L1B catalogue over Europe to generate maps of vegetation products from TOA radiance data at the continental scale. We generated both daily maps from daily OLCI images and also monthly averaged maps.

### 3.2 Spatial Analysis

**Figure 4:**
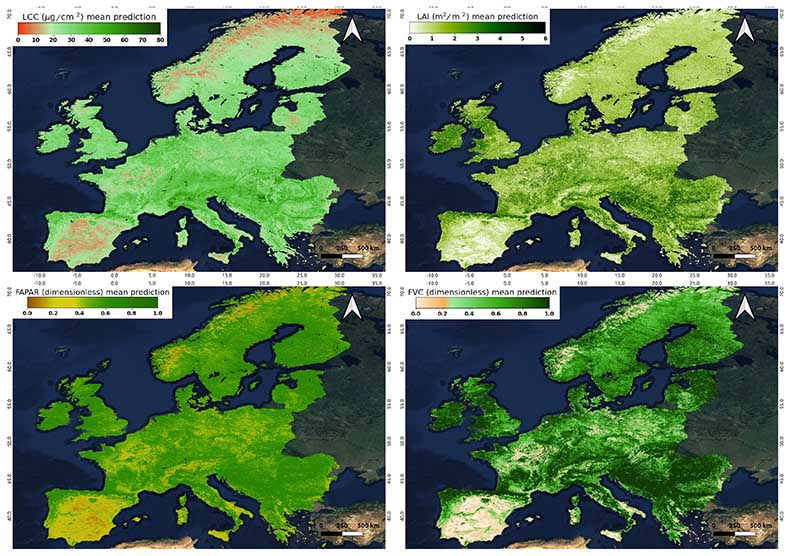
Monthly composite maps (July 2018) of vegetation traits produced by S3-TOA-GPR-1.0 models over Europe: LCC (top-left), LAI (top-right), FAPAR (bottom-left), and FVC (bottom-right).

**Figure 5:**
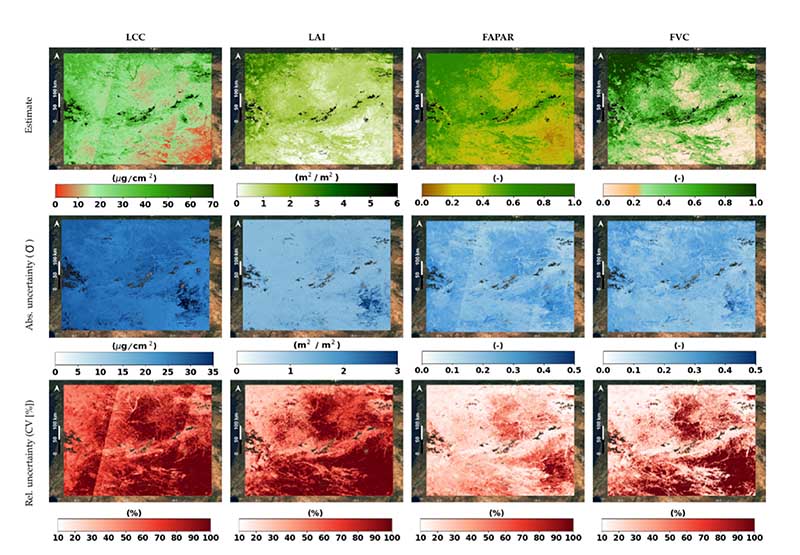
Retrieved daily maps, associated absolute uncertainty (*σ*) and relative uncertainty (CV) on 20 June 2019 over Iberian Peninsula (subset zoom-in indicated in Figure 2).

To demonstrate the mapping capability of our established S3-TOA-GPR-1.0 retrieval models, all four traits were spatially estimated for the whole of Europe (Figure 4), and for a specific part of the Iberian Peninsula (Figure 5). Figure 4 shows the monthly averaged maps from July 2018 for the different variables, obtained by applying S3-TOA-GPR-1.0 retrieval models to monthly averaged radiance data as explained in Section 2.3. The spatial distribution of LAI fits into the ranges defined by the global synthesis of Asner et al. [72], for different climates [73], ranging between 2 and 6 m^2^/m^2^ for temperate evergreen broad-leaved forests, 1-2 m^2^/m^2^ for boreal deciduous broad-leaved and evergreen needleleaf forests, as well as 0.5-2 m^2^/m^2^ for pastures and shrublands. Crops present a wide variety depending on the cultivation type and hence are hardly distinguishable at the actual spatial scale of 300 m. The other three vegetation traits present a similar spatial pattern, being correlated between each other. In the case of LCC, ranges of 30-80, 20-30 and 0-20 µg/cm^2^ are found over the same regions. FAPAR and FVC shift into similar general 0-1 scale, and also highlights the same spatial distribution.

Figure 5 shows examples of daily estimation maps and associated uncertainties, obtained for a S3 capture on 20 June 2019 over a part of Spain and Portugal (red square in Figure 2). Values of FVC and FAPAR range between 0 and 1 for areas shifting from dry to temperate climates respectively [73], with a corresponding uncertainty (1*σ*) generally lower than 0.15 provided by Equation (7) (see middle row of Figure 5). LCC and LAI estimates span between 0-70 µg/cm^2^ and 0-6 m^2^/m^2^, respectively, with maximum *σ* reaching around 35 µg/cm^2^ and 3 m^2^/m^2^. The maps in the bottom row of Figure 5 reflect the percentage deviation relative to the estimations, highlighting the areas with higher and lower reliable values. In this example, we detect the dark pixels in the South and center-top with maximum uncertainties for all the variables. Overall, the highest relative uncertainties are found associated to lower estimates, suggesting that the scarce or non-vegetated areas prevents our model from working in optimal conditions. Also, we find that FAPAR and FVC outperforms LCC and LAI models with generalized lower percentage deviations.

### 3.3 Temporal Analysis

**Figure 6:**
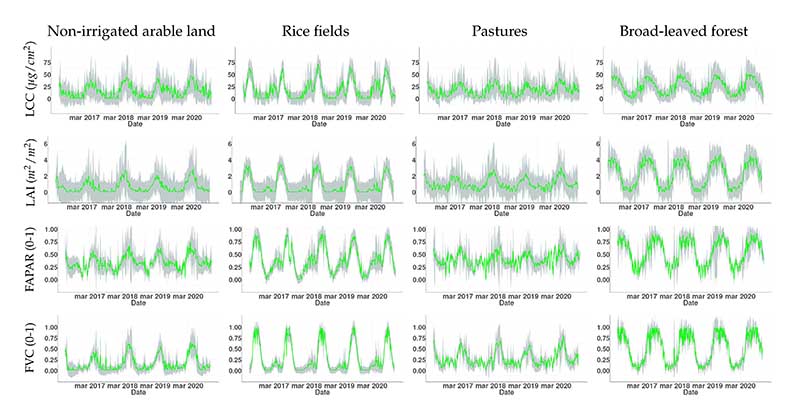
Time series of vegetation traits (LCC, LAI, FAPAR, FVC) over different land covers produced by the S3-TOA-GPR-1.0 models. The grey shaded colors present the uncertainty (*σ*) of the estimates (green). Original resolution of 300 m was used for spatial average calculations.

The temporal evolution of the four variables spatially averaged over the different classes of analyzed land covers is shown in Figure 6. We can assess the robustness of the models from the value of uncertainty (*σ*) associated to estimations (represented as grey shadows and green lines respectively). The *σ* account for deviations in estimations between both the prior and last predictor function states of our algorithm, informing about the accuracy of our model when facing real data. The graphs of LCC, LAI, FAPAR and FVC vary together in function of the land cover, with maximums of LCC close to 50, 70, 40, and 50 µg/cm^2^ for non-irrigated arable land, rice fields, pastures and broad-leaved forests, respectively. LAI reached values around 2, 3.5, 3 and 4 m^2^/m^2^ in the same order. FAPAR and FVC fluctuated similarly with maximum values up to 0.75, 0.90, 0.70, 0.96 (FAPAR), and 0.65, 0.95, 0.80 and 0.99 (FVC). In general, we observe a strong seasonality from the time series graphs, with peaks during spring and summer, and lowest values in winter. The best fits between estimates and *σ* were found for FVC. LCC also presented good results over rice fields. For the other combinations of land use classes and variables we observed more diverging *σ* values, especially for LAI.

Our estimates captured the phenology associated to each land cover. Non-irrigated arable land and pastures, being more dependent on rainfall, reach biomass maturity during spring on this regions, according to the national phenological calendar for different crops [74]. Maximum vegetative peaks around May are observed for non-irrigated arable land and pastures. Rice fields usually fully grow during July and August on the analyzed sites. Broad-leaved forests presented maximum values of biomass over longer periods (during spring and summer). The temporal profiles also reflected larger *σ* for periods with lower vegetation values, e.g., LCC and LAI over non-irrigated arable land and rice fields presented larger shadows on non-vegetative periods, suggesting again that these models (especially LAI) face difficulties dealing with scarce vegetation or bare soils.

### 3.4 Comparison and Validation Strategies

#### 3.4.1 Temporal Comparison against MODIS—MCD15A3H Products

**Figure 7:**
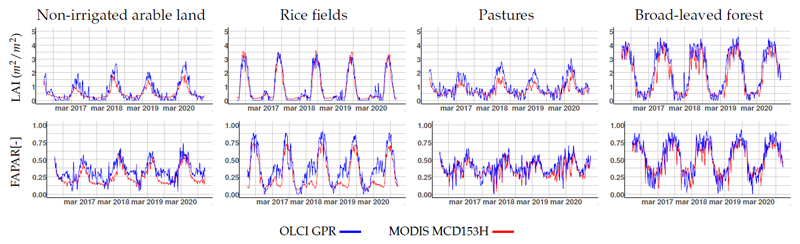
GPR method versus MODIS MCD15A3H product for LAI and FAPAR (rows) time series over following land covers: non-irrigated arable land, rice fields, pastures, and broad-leaved forests (columns). Original resolution of 300 m was used for spatial average calculations.

Temporal profiles generated by the S3-TOA-GPR-1.0 models are compared against the climatology of MODIS MCD15A3H LAI and FAPAR data, using the same time window of the retrieved S3-TOA-GPR-1.0 vegetation products (2016-2020). Evaluation on the different land covers are shown in Figure 7. Both products show similar seasonal patterns with different variability depending on the land cover type. Statistics summary of all variables and sites are listed in Table 4.

Closest agreement between both products were found on rice fields for LAI, and on broad-leaved forests for FAPAR, with minimum percentage differences between MCD15A3H and S3 TOA-GPR time series averages (ΔX in last column of Table 4) of 6.59% (LAI) and 7.27% (FAPAR). Conversely, the maximum differences were found for FAPAR over rice fields with ΔX of 33.27%. These differences are also observed in Figure 7, with absolute differences of FAPAR up to 0.25 during spring caused by a pronounced peak of the S3-TOA-GPR-1.0 estimates. For LAI, larges dissimilarities emerged on pastures, with ΔX of 27.7%, and absolute differences up to 1.5 m^2^/m^2^ on the early summer of 2018. In the case of LCC and FVC, MODIS reference products were not available and therefore the OLCI results are summarized in Table 4. Maximum values of 4.61 m^2^/m^2^ (LAI), 0.94 (FAPAR) and 0.97 (FVC) were found for broad-leaved forests, while the peak of 67 µg/cm^2^ (LCC) is found over rice fields. Both S3-TOA-GPR-1.0 and MCD15A3H-MODIS time series profiles were produced by applying the time series gap-filling described in Section 2.3.

#### 3.4.2 Spatial Comparison against CGLS Products

**Figure 8:**
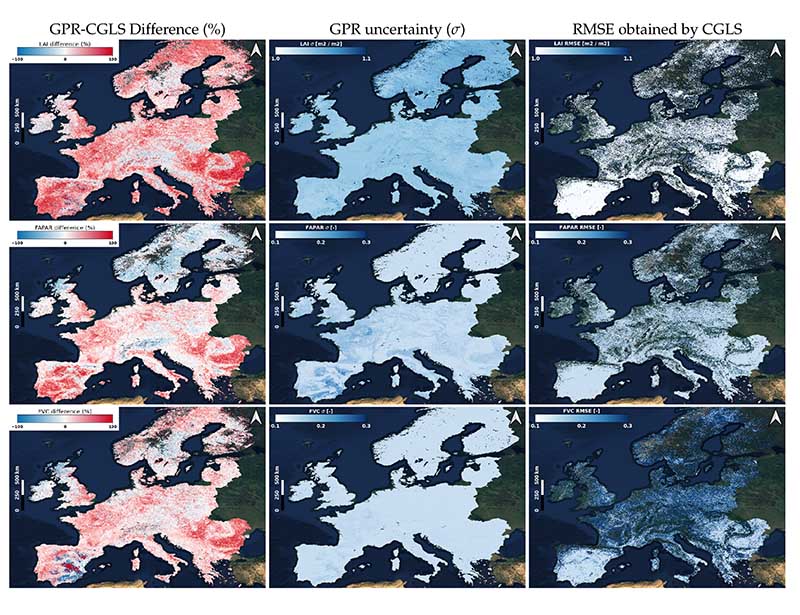
Comparison maps between CGLS and S3-TOA-GPR-1.0 models for a monthly averaged composed map between 4 March 2019 and 20 November 2019. In columns: maps of GPR-CGLS percentage differences (**left**), uncertainty (*σ*) obtained by GPR (**middle**) and RMSE obtained by CGLS (**right**). In rows: maps of LAI (**top**), FAPAR (**middle**) and FVC (**bottom**).

Figure 8 shows the spatial intercomparison against reference products provided by CGLS. The maps in Figure 8 show the spatial distribution of the differences. They suggest that the CGLS products provide higher values for some scarcely vegetated areas in the Iberian Peninsula in the case of LAI and FVC, and also in dense vegetated regions around the Alps and parts of Italy and Carpathian Montane forests. On the Scandinavian Peninsula, we also remark that the S3-TOA-GPR-1.0 models provide lower values, especially for FAPAR. Apart from those, our S3-TOA-GPR-1.0 models generally provide higher values. Figure 8 also demonstrates maps of GPR uncertainties (*σ*) and RMSE of CGLS products. Stronger uncertainties emerge in the same areas (Iberian Peninsula) where highest underestimation of our products occurred for LAI and FVC. In Scandinavia, we observe larger *σ* than RMSE for LAI but the opposite for FVC. Also, in the Northeast of France we find a prominent surface with curved-shape, corresponding to cereal crop lands according to [62], with larger *σ* in all the three maps. In general, both RMSE and *σ* shift into similar ranges for remaining areas, although it should be taken into consideration that the RMSE presented a substantial percentage of missing pixels.

#### 3.4.3 Validation against VALERI Ground Data

**Figure 9:**
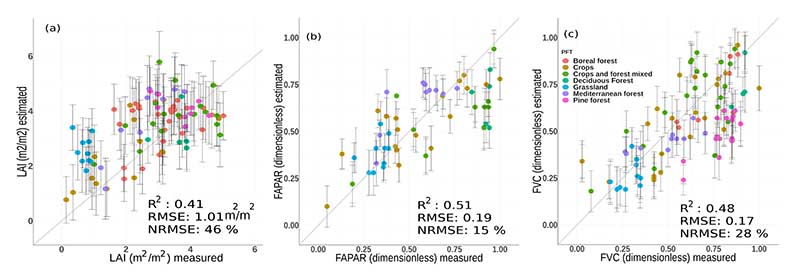
Scatter plots of predicted vegetation traits LAI (**a**), FAPAR (**b**) and FVC (**c**) by OLCI GPR models vs. interpolations based on ground measurements of the VALERI campaigns. The vertical bars show the obtained estimated uncertainty (*σ*).

Figure 9 demonstrates the goodness-of-fit obtained by the S3-TOA-GPR-1.0 models (trained with 18 bands) against interpolated ground measurements of the VALERI campaign. The plots indicate a moderate correlation for all three variables, with highest accuracy for FAPAR and FVC estimates (NRMSE of 15% and 28% respectively), and lowest for LAI (NRMSE of 46%). The samples were colored according to different land cover categories including crops, forests, grasslands and mixed types. For all three variables, heteroscedastic behaviour of residuals can be observed and nonlinearity is present, showing saturation of higher values. In the case of LAI, overestimation was observed in particular for grasslands and crops, with estimated values between 1.8 and 3.3 m^2^/m^2^ corresponding to measured values in a range between 0.3 and 0.95 m^2^/m^2^. The samples belonging to the other land covers are more disperse along the 1:1-line. The FAPAR scatter plot shows that the samples per class are more compactly distributed compared to LAI. It must be remarked that the samples belonging to the Mediterranean forest class result in constant estimates values around 0.7 for a range of measured values between 0.5 and 0.75. Conversely, the estimations of deciduous forests present values between 0.5 and 0.8 while linked to constant measured values around 0.9. The scatter plot of FVC shows a good fit along the 1:1-line, highlighting in this case underestimations of the pine forest samples.

## 4 Discussion

GEE became a popular platform in facilitating research activities in the field of vegetation traits mapping and monitoring. At the same time, despite an increasing number of studies exploring GPR models in the remote sensing domain [31,49,59,75–77], the integration of GPR in GEE to produce spatiotemporal information of vegetation still remained to be explored. Therefore, in this study we presented a workflow for monitoring vegetation traits at continental scale by taking advantage of the TOA S3-OLCI collection available in the GEE cloud platform. Hereafter, we discuss the obtained results of our S3 TOA-GPR models, in relation to the spatiotemporal consistency (Section 4.1), validation strategies (Section 4.2), uncertainties and limitations of the used models (Section 4.3), and future perspectives derived from our study (Section 4.4).

### 4.1 Spatiotemporal Consistency

Our workflow expands previous efforts to implement GPR retrieval models in GEE by exploiting for the first time OLCI TOA data in a hybrid approach. In addition, our analysis was extended to four essential vegetation traits and mapping was performed at a large spatial scale. In general terms, the produced traits retrievals responded consistently in space and time. Specifically, the generated monthly averaged maps (Figure 4) are comparable to reference studies showing similar spatial patterns as the obtained by PROBA-V Collection CGLS products [64]. At the spatial scale, the obtained trait maps at a European level (Figure 4) revealed ecoregions with the four variables presenting values within expected ranges. For instance, LAI values of different plant functional types were well simulated by the S3-TOA-GPR-1.0 models, with outputs ranging from 0 to 6 m^2^/m^2^ as function of the biome. Given the dependency between LAI, FAPAR and FVC [78], but also LCC, we observe a similar spatial pattern for all variables across Europe (see Figure 4), with FAPAR and FVC quantified over the full range of 0-1. The spatial behaviour of LCC was found closely related to LAI with peaks mainly reached over denser vegetated regions.

For a closer inspection, when focusing on the four traits over the Iberian Peninsula (Figure 5), we clearly identified permanent densely vegetated areas with maximum values in the north (Cantabrian Mountains) or in the central-west mountainous regions. Also, the maps coincidentally reflect the lower values at the south-east corresponding to bare soils and crops, mainly vineyards and non-irrigated lands according to the CORINE Land Cover Classification [62]. Our results were in agreement with those of Pipia et al. [47], highlighting the same spatial behaviour for LAI from Sentinel-2 imagery. The authors also applied GPR models for predictions at dates matching the time windows of our monthly composition maps (see Figure 4b), using as inputs Sentinel-2 L2A data. In the same figure we also provide associated uncertainty information in absolute and relative terms, which helps to evaluate robustness and fidelity of retrieval models [75]. Although the patterns are spatially similar, LCC and LAI retrievals are subject to higher uncertainty than FAPAR and FVC, which confirms the theoretical model results (see Figure 3). Thanks to this Bayesian capability of GPR, we can apply the S3-TOA-GPR-1.0 models to different locations and large heterogeneous scenes, and directly obtain feedback about the estimation quality through the associated uncertainties. Although alternative retrieval strategies may perform similarly in terms of accuracy and processing speed, they all lack this outstanding capability. With uncertainty estimates, not only valuable additional information is obtained next to validation relying on in situ data, it also can be used for masking out of uncertain areas, e.g., at a given threshold [79,80].

The presented monthly averaged maps underline the utility of temporally-aggregated data for analysis, independent of particular daily conditions, and without spatial gaps e.g., due to cloud cover. In this context, we tested monthly medians and averages and applied both over S3-OLCI L1B radiance data per pixel. Both statistics are suitable for composing maps and resulted in similar outputs for July 2018. Nevertheless, if outliers are present in the time composition windows, the medians are the more suitable option to compensate anomalous values.

In this work we chose to process the gap-filled images with a monthly interval for sake of demonstration. Yet, the temporal aggregation can be just as well applied to other time intervals for monitoring specific events. For instance, we can assess the impact of extreme temperatures and droughts on vegetation by using FAPAR estimations on shorter time scales [81]. Extreme weather events, increasing in frequency in the context of climate change, can lead to degradation processes on fragile ecosystems, such as observed in the cork oak forests in the Mediterranean basin [82]. Likewise, outbreaks of plagues on forest trees or crops can be noticed on short time intervals [83,84]. Furthermore, efficiency of croplands monitoring depends upon the availability of timely and accurate data, which can be delivered by our methodology. Thus, real time information about crops or forest status is crucial to commit adequate decisions and to ensure an optimal management of environment and agricultural resources [85,86].

At the temporal scale, Figure 6 illustrates the expected seasonal cycles over the five recorded years. The time series were obtained from spatially averaged points over time, of both estimations and deviations (*σ*). With this strategy we aimed to demonstrate the capabilities of this workflow to also capture the evolution of different vegetated surfaces including the associated uncertainties. Such data streams can serve diverse applications, for instance tracking the conservation state of landscapes comprehensively [87], quantifying tree cover forest changes based on the analysis of time series trends per pixel [88], or analyzing ecological vulnerability and phenological responses [89]. The temporal analysis here addressed also reveals the utility in the context of cropland phenology studies, e.g., through the calculation of land surface phenology (LSP) metrics, such as start or end of season [90–92].

### 4.2 Product Intercomparison with Validation Data Sets

One of the major bottlenecks in vegetation retrieval studies is probably the limited availability of trustful validation data sets. For our study, direct in situ ground measurements were missing, collected with the required sampling strategy to represent the coarse 300 m OLCI pixels. To circumvent this, we chose three different products for direct and indirect evaluation of the S3-TOA-GPR-1.0 models. Note also that none of the reference products included LCC, hence a comparison for this variable is lacking. In the following, we comment our findings when comparing and validating our results against the MODIS MCD15A3H, the CGLS products, and the data coming from the VALERI project, respectively.

The temporal intercomparison analysis against the MODIS MCD15A3H collection revealed a generally close agreement between both products. Largest deviations occurred on rice fields in spring, and over non-irrigated arable land in summer. These differences were mainly reached during the dormant periods.

When contrasting our composition maps against the reference CGLS products we obtained closer agreement for FAPAR and FVC than for LAI (Figure 8). This reflects the higher theoretical retrieval results obtained for the two products (see Figure 3). Additionally, the comparative maps highlighted differences depending on the land cover type, revealing that the model's performances are not spatially homogeneous. This point is also confirmed by the analysis of uncertainty carried out at the scale of the Iberian Peninsula (Figure 5), where we detected least consistent results when analyzing bare soils, with stronger relative deviations with regards to estimation values.

Concerning the direct validation against the VALERI products, we observe a moderate to good retrieval performance for the three variables LAI, FAPAR and FVC. A few aspects may limit the informative value of this comparison study. First, a resampling strategy based on interpolations was required to compare both products at the same spatial resolution, i.e, from 20-30 m (SPOT/Landsat) to 300 m (S3). Second, OLCI L1B TOA radiance was simulated using Landsat ETM data due to missing availability of S3 scenes before 2016. In relation to this, the models were built using only 18 from 21 bands due to lack of spectral information below 490 nm of Landsat ETM. Despite all, we decided to consider the VALERI data set for validation as it presents a unique data base for testing our S3-TOA-GPR-1.0 retrieval models: VALERI encompassed a network of sites (and a methodology) to validate medium spatial resolution satellite products over terrestrial surfaces [66]. For this purpose, field data were taken over a variety of plant functional types from different geographical areas, and in addition, upscaled products for validating satellite images were provided. These two aspects render the VALERI data set ideal for the spatial context of our work.

### 4.3 Study Limitations and Challenges

Identified limitations and challenges are discussed next. (1) We first elaborate the assumptions and parametrization of the used RTMs, i.e., SCOPE and 6SV, in order to design the optimal training data set. Following, (2) uncertainties related to sub-pixel spectral heterogeneity is commented. Further, (3) the impact of seasonality on our results is analyzed, and finally, (4) the difficulties encountered when implementing the S3-TOA-GPR-1.0 models in GEE are discussed.

#### 4.3.1 Assumptions and Parametrization of SCOPE and 6SV

The S3-TOA-GPR-1.0 models were trained with simulated data coming from leaf-canopy-atmosphere RTMs. We used the SCOPE model [93] to generate a canopy-level data set, which was then upscaled to TOA with atmospheric transfer functions coming from 6SV according to Verrelst et al. [33] and Estévez et al. [49]. Simulations of the SCOPE model are based on 1D turbid medium assumptions of the canopy. Hence, the trained models better approximate homogeneous vegetation stands, such as crops or grassland, than heterogeneous, such as forests. Nonetheless, the influence of complex 3D canopy structures becomes less evident at the spatial resolution of S3 with 300 m [94,95] compared to higher spatial resolution products. Comparing our LAI and FAPAR retrievals with the MODIS MCD15A3H products, which are inferred from a 3D RTM, we even found higher consistency over (broad-leaved) forests than crops (i.e., non-irrigated arable land). These results suggest that (regular) seasonality plays a more important role than the complexity of the underlying model when comparing time series of MODIS MCD15A3H and S3-TOA-GPR-1.0 models. Despite consistent results it is worth mentioning that limitations of the 1D simulations have been widely reported, for instance due to the light obstruction by clumped foliage, tree crowns, branches, and shoots, not being quantified, leading to biased LAI retrieval results [6,96]. It must also be remarked that the theoretical performance of the established models revealed heteroscedasticity, which may limit the retrieval accuracy in particular of higher variable values.

Further, we assumed a Lambertian surface to upscale the TOC reflectance towards TOA radiance [33,56]. In reality, vegetation canopies may not be optimal Lambertian diffusers. Uncompensated atmospheric scattering caused by the Lambertian assumption may systematically introduce uncertainty into the retrieval results [97]. On the other hand, assuming a Lambertian approximation renders the computation more feasible by reducing the required size of the simulated data sets used for training. Moreover, the Lambertian approximation only introduces a small error for near nadir observations for situations approaching the hot spot region [97–99].

Finally, among the main challenges in the context of vegetation traits mapping at continental scale when relying on hybrid models is the design of a representative training data set, which should cover a wide variability of different vegetation states. Besides RTM simulations spreading over a vast number of parameter combinations, real measurements were taken into account from the S3 scenes to provide a realistic training data set valid for a variety of vegetated and non-vegetated states. The uncertainty estimates (*σ*) provided by the S3-TOA-GPR-1.0 models can be used to directly assess the impact associated to the selected training inputs: the kernel-based distance between the OLCI observations and the training samples is reflected on the *σ* values [36].

#### 4.3.2 Sub-Pixel Spectral Heterogeneity in Transitional Vegetation Areas

With a spatial resolution of 300 m for the nominal orbit, subpixel heterogeneity of one OLCI pixel will be present for many landscapes. If the assumptions of the training data set are not met by spectra coming from heterogeneous surfaces, the performance of the model will decline. Although maps show consistent patterns, it is likely that heterogeneous surfaces are suboptimally estimated. According to De Grave et al. [6], a way to deal with pixel heterogeneity is to train a model including synthetic mixed spectra composed of a linear combination of pure vegetated spectra and pure bare soil spectra [100–102]. Additionally, although we filtered out bright pixels using the quality flag, still low percentage of cloud contamination within a pixel may be present, causing some noise. To deal with this topic, we added external spectra coming from heterogeneous surfaces, as observed directly from OLCI, assigning vegetation traits values coming from external sources (MODIS MCD153H product). In future work, filtering pixels in function of their homogeneity appears as a solution for improving results. In this regard, observations at higher scale resolution, coming from instruments such as Sentinel-2 or Landsat-8 ETM+ may contribute to homogeneity estimations.

#### 4.3.3 Time Series and Impact of Seasonality

Inspection of the temporal profiles obtained by LCC, LAI, FAPAR, and FVC reveal performance differences depending on the date of the year. For instance, in the case of LAI for rice fields (Figure 6) we can observe that between November and March the associated uncertainty (*σ*) increases. A possible explanation for this is that the spectral response of the land cover properties varies along the year (e.g., due to irrigation), with our model responding differently to the diverse states. On the other hand, during winter the quantity of cloudy days generally increases [103,104]. We applied gap-filling to the time series to interpolate missing data, but the performance of this technique decreases in function of the time-length of gaps due to missing data [27,105].

Another related aspect affecting estimations throughout the year is the role of varying sun-target-sensor geometry in combination with latitude. Accordingly, the S3-TOA-GPR-1.0 models were trained to cover a variety of theoretical conditions, for instance sun zenith angles in a range of 20° to 40° were considered, as the values most commonly reached over European latitudes most part of the year at the crossing time of S3. We used a sun position calculator accessed at http://www.sclartcpc.ccm/sclar-crbit on 1 May 2021 to determine this range. The reason to constrain the solar geometry to this range was to find a balance for the model to stay reasonably lightweight for the proper running in GEE. This implies that for ranges beyond the training limits, or for particular combinations (e.g., on winter over northern latitudes), the model will respond less optimally.

#### 4.3.4 GEE Processing

The GEE platform excels by its capacity to provide a parallel computation service allowing to run tasks by using multiple CPUs connected in the cloud [43]. Nevertheless, a limited quota of memory usage is established for public usage, restricting in particular the operations on matrices of big size dimensions used in this work. This results in reduced dimension data sets having to cover a broad variability in training data. We addressed these limitations by ranging input variables over all possible combinations, reducing the data set by a random function and then mixing with spectra taken from real scenes over different locations and dates. To assess the performance of the trained model, we introduced the uncertainty calculations in GEE as a novelty allowing to filter the outputs in function of their intrinsic consistency.

It must also be remarked that when using the OLCI L1B collection through GEE directly, a mismatch on values over specific locations relative to original data provided by Copernicus Open Data Hub Services has been reported due to use of a different coordinate system (i.e., tie points vs. geo-coordinates) [106]. In our case, these local mismatches are not measurable when working at large scale. We kept the OLCI native spatial resolution (300 m) for the traits mapping. Nevertheless, when processing time series over large areas (e.g., Spain), eventually, lower resolutions will be applied for aggregation operations (e.g., spatial average) for reasons of performance and processing time. In our case, the missing information masked into lower resolution images resulted into time series providing lower values. This is explained as native resolution pixels are averaged through a pyramidal aggregation process when changing the scale (e.g., continental), leading to smoothed values [43].

### 4.4 Opportunities for Future Work

In the upcoming years, new generation satellites and cloud-based processing technologies will stimulate advances in quantifying fundamental vegetation traits. In this context, the S3-FLEX mission will deliver complementary information of surface fluorescence, reflectance and temperature, allowing to monitor the photosynthetic activity of the actual terrestrial vegetation across the globe at a spatial resolution of 300 m [4]. The establishment and assessment of accurate models for spatiotemporal large scale mapping of vegetation traits using these unprecedented data streams remained still open. Hereby, cloud-based computing provides promising opportunities, avoiding complex processes for obtaining the input data (e.g., data acquisition and managing databases) or processing supercomputers [43]. Due to the memory restrictions of GEE, lightweight models have to be guaranteed as the GPR algorithms tend to be exigent in terms of memory size. To circumvent this limitation, active learning (AL) methods can be employed. AL methods select only most relevant samples from a training data pool, enhancing efficiency of the algorithm training process [107,108]. Alternatively, the implementation of other ML methods in the GEE environment may open possibilities when working with large training data sets. A study of global scale traits mapping using random forests in GEE is provided by Campos-Taberner et al. [46]. While random forests can become an appealing alternative processing technique, the provision of associated uncertainty estimates is still missing.

The workflow presented here can contribute to build multi-annual or even multidecadal time series of regional phenological information at a large spatial scale. The inspection of time series profiles allows to study ecological changes over large areas, such as drought, fires or land use changes [109,110]. In this respect, an approach to extend the OLCI data catalogue back in time was proposed by Pipia et al. [111], based on the use of multi-output Gaussian processes regression (MOGPR). This method relies on the same mathematical principles described in this work, but then extending for predicting multiple variable outputs from multiple independent input time series data streams. In practical terms, as demonstrated by Pipia et al. [111], this concept opens the opportunity to reconstruct temporal patterns making use of data streams coming from other sensors, including historical time series data. In our case, given that the currently covered years of the OLCI catalogue is limited to study decadal processes, MOGPR can be used to reconstruct OLCI-like data guided by MODIS data collection for at least two decades back in time. Follow up research is foreseen to explore MOGPR models in the GEE platform.

Data streams of the fundamental vegetation traits can further be assimilated into models for estimating global gross primary productivity [112]. Recently, this was demonstrated also with the contribution of fluorescence estimates [113], and is foreseen to be developed within the S3-FLEX tandem mission concept. Consequently, the vegetation retrieval models presented here are expected to contribute to assimilation processing chains aiming to quantify photosynthetic activity once FLEX is launched and starts transmitting data [114]. A key requirement regarding assimilation systems is the provision of uncertainties along with the satellite derived products to propagate through the assimilation trajectory [115], which our GPR models provide.

All in all, the unprecedented processing capabilities of the GEE environment will increase our understanding regarding speed of vegetation change and dynamics across diverse ecosystems [2]. Provision of vegetation traits maps in real time may support related management decisions and hence will be valuable tools for decision makers. The applicability of our work on a global scale relying on vegetation inputs can be wide, e.g., carbon balance estimations [116,117], integration in multi-instrument analysis and synergies [118] or assessment of photosynthetic activity in the context of the upcoming FLEX mission [4,7].

## 5 Conclusions

Global vegetation monitoring by means of large scale satellite sensors is fundamental for understanding multiple ecological processes and supporting carbon sink quantification. In our study we presented a retrieval framework to generate monthly averaged maps and spatially averaged time series of the four vegetation traits LCC, LAI, FAPAR and FVC at a continental scale. To achieve this, we implemented pretrained GPR models in GEE, named as S3-TOA-GPR-1.0, to enable the processing of the entire collection of S3 OLCI top-of-atmosphere radiance (L1B) images provided over Europe. Overall mapping was achieved in a good accuracy as validated against temporal (MODIS MCD15A3H), spatial (CGLS) and interpolated in situ (VALERI) reference products. Theoretical validation and comparison of our S3-TOA-GPR-1.0 models to the three reference products suggested a better match for FAPAR and FVC than for LAI and LCC.

In summary, our proposed method offers the following main advantages:

- The atmospheric correction step is avoided due to direct processing of S3 TOA images, optimizing also computational running time.
- GPR models provide uncertainties along with the predictions allowing to evaluate the robustness, consistency and fidelity of retrieval models.
- Implementation of traits retrieval model into the GEE platform enables large scale processing of multiple trait maps.

We conclude that the workflow presented here provides a pathway towards operational mapping of quantitative vegetation products at the continental or even global scale. It is foreseen that the obtained estimates will further support the correct interpretation of sun-induced fluorescence obtained from observations of the upcoming Earth Explorer 8 within the S3-FLEX tandem mission. This synergistic data interpretation may enhance our understanding about ecological processes and increase the ability to face future challenges in the context of global climate change.

## Supplementary Material

Appendix

## Figures and Tables

**Table 1:** Biochemical leaf and canopy structure variables used for SCOPE simulations. See [6] for details about default values.

Variable	Distribution	Min	Max	Mean	SD
Leaf structure & biochemistry					
N (Leaf structure parameter [-])	Gaussian	1	2.7	1.5	0.5
LCC (Chlorophyl a,b content, µg/cm^2^)	Uniform	0	95.6	-	-
Cxc (Carotenoid content, µg/cm^2^)	Gaussian	0	20	10	10
Cdm (Dry matter content, g/cm^2^)	Gaussian	0.002	0.02	0.005	0.003
Cw (Leaf water content, cm)	Gaussian	0.005	0.035	0.012	0.006
Canopy structure					
LAI (Leaf Area Index, m^2^/m^2^)	Uniform	0	7.0	-	-
LIDF (Leaf Inclination, rad)	Uniform	—1	1.0	-	-
Soil					
SMC (Soil Moisture Content, %)	Gaussian	5	55	25	12.5
BSM Brightness	Gaussian	0	0.9	0.5	0.25
BSM Lat (°)	Gaussian	20	40	25	12.5
BSM Long (°)	Gaussian	45	65	50	10
Geometry					
SZA (Sun Zenith Angle, °)	Uniform	20	40	-	-
OZA (Observation Zenith Angle, °)	Uniform	—10	10	-	-
RAA (Relative Azimuth Angle, °)	Constant	180	180	-	-

**Table 2:** Range of 6SV input variables used for the simulations of the atmospheric transfer functions.

Model Variables	Units	Range
Atmospheric variables: 6SV		
O_3_ Column concentration	[amt-cm]	0.25-0.35
Columnar Water Vapor	[g·cm^-2^]	0.4-4.5
Aerosol Optical Thickness	unitless	0.05-0.5
Angstrom coefficient	unitless	0.05-2
Henyey-Greenstein asymmetry factor	unitless	0.6-1

**Table 3:** Overview of the three data sets used for direct and indirect validation of vegetation traits models, including spatial and temporal resolutions, dimensions, sensors, source algorithms, strategy of presented validation and targeted variable. NDVI: Normalized Difference Vegetation Index.

Validation Source	Spatial Resolution of Source	Temporal Resolution of Source	Validation Dimension	Sensor	Source Algorithm	Validation Strategy	Target Variables
MCD15A3H - MODIS	500 m	4 days	spatiotemporal	MODIS	empirical relationship with NDVI. RTM based LUTs	time series differences	LAI, FAPAR
CGLS Vegetation V1.1	300 m	composition maps: 10 days, 1 month, season range	spatiotemporal	PROBA-V/OLCI	ANN	percentual differences	LAI, FAPAR, FVC
VALERI high resolution biophysical maps	20 m	time range of ground measurements: i.g., 1 day, 2 days	space	SPOT-HRVIR m	empirical transfer function between ground measurements and high resolution spectral data	scatter plots	LAI, FAPAR, FVC

**Table 4:** Summary statistics over temporal window (April 2016-November 2020) for LAI, FAPAR, LCC and FVC, refered to CLC areas. LAI and FAPAR of the S3-TOA-GPR-1.0 models are compared against MODIS MCD15H products with percentage differences of time series means (ΔX%).

MODIS LAI/FAPER					OLCI LAI/FAPER		
Variable/Site	$\bar{\text{X}}$	SD	MAX	$\bar{\text{X}}$	SD	MAX	AX%
LAI/BF1	1.91	0.14	4.26	2.28	1.49	4.61	16.38
LAI/NIAL	0.52	0.41	1.89	0.71	0.73	3.01	26.14
LAI/RF	0.85	1.07	3.63	0.91	1.13	3.50	6.59
LAI/P	0.77	0.37	2.39	1.06	0.68	3.03	27.70
FAPAR/BF1	0.51	0.20	0.81	0.55	0.24	0.94	7.27
FAPAR/NIAL	0.26	0.12	0.60	0.35	0.13	0.74	24.65
FAPAR/RF	0.27	0.22	0.75	0.40	0.25	0.91	33.27
FAPAR/P	0.36	0.11	0.68	0.40	0.12	0.70	8.90
**OLCILCC**					**OLCI FVC**		
**Site**		**X**	**SD**	**MAX**	**X**	**SD**	**MAX**
BF1		24.14	10.13	59.92	0.55	0.10	0.97
NIAL		10.44	10.15	34.63	0.22	0.10	0.81
RF		15.85	8.18	67.04	0.29	0.08	0.96
P		11.73	9.79	31.58	0.28	0.10	0.83

BF1: broad-leaved forest; NIAL: non-irrigated arable land; RF: rice fields; P: pastures.

## Data Availability

The following link contains a repository with the codes and models used in this paper for spatiotemporal mapping of vegetation, open available for the community: https://github.com/psreyes/S3_TOA_GPR_1.git (accessed on: 8 January 2022).
