# Supplementary material for: Quantifying Fundamental Vegetation Traits over Europe Using the Sentinel-3 OLCI Catalogue in Google Earth Engine
Source: Remote Sens (Basel). Author manuscript; Available in PMC 2022 Aug 24. (PMC7613398; doi:10.3390/rs14061347)
Supplement: Appendix [file EMS152682-supplement-Appendix.pdf]

Appendix A

Table A1. Validation campaigns and products offered by the VALERI network.

Country	Site	Boundaries Coordinates	Land Cover	Date of Ground Data Collection	Variables	Interpolated Biophysical Map Spatial Resolution (m)	SPOT Image for Interpolation Transfer Function	
							Aquisition Date	SZA
Belgium	Sonian forest	50.78°N–50.77°N 4.38°E–4.41°E	forest	2004/06/21–2004/06/22	LAI, FAPAR, FVC	20	2004/07/28	34.58
England	Chilbolton	51.19°N–51.14°N 1.47°W–1.43°W	crops and forest	2006/06/14–2006/06/17	LAI, FVC	10	2006/07/10	28.90
Estonia	Jarvselja	58.31°N–58.29°N 27.24°E–27.26°E	boreal forest	2007/07/18–2007/07/19	LAI, FVC	20	2007/06/16	35.5
Estonia	Jarvselja	58.31°N–58.29°N 27.23°E–27.26°E	boreal forest	2002/06/24–2002/06/30	LAI, FVC	20	2002/07/13	36.83
Estonia	Jarvselja	58.31°N–58.29°N 27.23°E–27.26°E	boreal forest	2005/06/28–2005/07/01	LAI, FVC	20	2005/06/20	35.64
France	Les Alpilles	43.82°N–43.81°N 4.70°E–4.71°E	crops	2002/07/22–2002/07/23	LAI, FAPAR, FVC	20	2002/07/20	49.04
Romania	Fundulea	44.42°N 26.56°E	crops	2003/05/24	LAI, FAPAR, FVC	10	2003/05/31	24.43
Spain	Barrax	39.04°N 2.21°E	cropland	2007/07/01	LAI, FAPAR, FVC	20	2003/07/03	22.11

Table A1. *Cont.*

Country	Site	Boundaries Coordinates	Land Cover	Date of Ground Data Collection	Variables	Interpolated Biophysical Map Spatial Resolution (m)	SPOT Image for Interpolation Transfer Function	
							Aquisition Date	SZA
Germany	Gilching	48.10°N–48.08°N 11.30°E–11.32°E	crops and forests	2002/07/17–2002/07/19	LAI, FAPAR, FVC	20	2002/07/08	29.16
France	Nezer	44.62°N–44.56°N 1.09°W–1.04°W	pine forest	2002/04/23	LAI, FAPAR, FVC	20	2002/04/21	34.28
France	Puechabon	43.74°N–43.72°N 3.63°E–3.65°E	mediterranean forest	2001/06/11–2001/06/15	LAI, FAPAR, FVC	20	2001/06/12	25.94
France	Larzac	43.95°N–43.94°N 3.10°E–3.12°E	grassland	2002/07/01–2002/07/03	LAI, FAPAR, FVC	20	2002/07/12	27.39
